# Formulation, Pharmacokinetic, and Efficacy Studies of Mannosylated Self-Emulsifying Solid Dispersions of Noscapine

**DOI:** 10.1371/journal.pone.0146804

**Published:** 2016-01-12

**Authors:** Terrick Andey, Apurva Patel, Srujan Marepally, Mahavir Chougule, Shawn D. Spencer, Arun K. Rishi, Mandip Singh

**Affiliations:** 1 Department of Pharmaceutical Sciences, School of Pharmacy, Massachusetts College of Pharmacy and Health Sciences University, 19 Foster Street, Worcester, MA, United States of America; 2 Department of Pharmaceutics, College of Pharmacy and Pharmaceutical Sciences, Florida A&M University, 1520 South Martin Luther King Jr. Blvd., Tallahassee, FL, United States of America; 3 Institute for Stem cell biology and Regenerative Medicine (inStem), National Centre for Biological Sciences (NCBS), Bangalore, India; 4 Department of Pharmaceutical Sciences, Daniel K. Inouye College of Pharmacy, University of Hawaii at Hilo, 200 W. St., Hilo, HI 96720, United States of America; 5 Department of Oncology, Wayne State University, Detroit, MI, United States of America; Roswell Park Cancer Institute, UNITED STATES

## Abstract

**Purpose:**

To formulate hydroxypropyl methylcellulose-stabilized self-emulsifying solid dispersible carriers of noscapine to enhance oral bioavailability.

**Methods:**

Formulation of noscapine (Nos) self-emulsifying solid dispersible microparticles (SESDs) was afforded by emulsification using an optimized formula of Labrafil M1944, Tween-80, and Labrasol followed by spray-drying with hydroxypropyl methylcellulose (HPMC), with and without mannosamine (Mann-Nos_SESDs and Nos_SESDs respectively); self-microemulsifying liquid dispersions (SMEDDs) with and without mannosamine (Mann-Nos_SMEDDs and Nos_SMEDDs respectively) were also prepared. SMEDDs and SESDs were characterized for size, polydispersity, surface charge, entrapment efficiency, in vitro permeability, in vitro release kinetics, and oral pharmacokinetics in Sprague-Dawley rats (10 mg/kg p.o). The antitumor efficacy of Mann-Nos_SESDs on the basis of chemosensitization to cisplatin (2.0 mg/kg, IV) was investigated in a chemorefractory lung tumor Nu/Nu mouse model up to a maximal oral dose of 300 mg/kg.

**Results:**

The oil/surfactant/co-surfactant mixture of Labrafil M1944, Tween-80, and Labrasol optimized at weight ratios of 62.8:9.30:27.90% produced stable self-microemulsifying dispersions (SMEDDs) at a SMEDD to water ratio of 1–3:7–9 parts by weight. SMEDDs had hydrodynamic diameters between 231 and 246 nm; surface charges ranged from -16.50 to -18.7 mV; and entrapment efficiencies were between 32 and 35%. SESDs ranged in size between 5.84 and 6.60 μm with surface charges from -10.62 to -12.40 mV and entrapment efficiencies of 30.96±4.66 and 32.05±3.72% (Nos_SESDs and Mann-Nos_SESDs respectively). Mann-Nos_SESDs exhibited saturating uptake across Caco-2 monolayers (P_app_ = 4.94±0.18 × 10^−6^ cm/s), with controlled release of 50% of Nos in 6 hr at pH 6.8 following Higuchi kinetics. Mann-Nos_ SESDs was 40% more bioavailable compared to Nos_SESDs; and was effective in sensitizing H1650 SP cells to Cisplatin in vitro and in an orthotopic lung tumor model of H1650 SP origin.

**Conclusions:**

Mannosylated noscapine self-emulsifying solid dispersions (Mann-Nos_SESDs) are bioavailable and potentiate the antineoplastic effect of cisplatin-based chemotherapy in cisplatin-resistant NSCLC.

## Introduction

Noscapine, a low toxicity, naturally-occurring benzylisoquinoline alkaloid is associated with anticancer activity [1,2]. The mode of action of noscapine’s anticancer activity is polymerization and stabilization of microtubules [3]; and when administered in combination with conventional chemotherapy, it potentiates the induction of cell death [4]. However, the prospect of noscapine as an effective anticancer therapy in the clinic remains unknown [5,6]. That noscapine, a lipophilic compound (LogP ~2.6) with moderate aqueous solubility (solubility ≤ 0.05 mg/mL) should suffer from limited oral bioavailability is underpinned by a short half-life stemming from extensive hepatic metabolism, as is common with opioids [7,8]. Noscapine’s anticancer activity therefore, necessitates a high oral effective dose (ED_50_ 300–600 mg/kg), thereby, limiting its translational utility due to potential adverse reactions [9,10]. There has consequently been much interest in nanoparticle encapsulation of noscapine as a means of overcoming reduced plasma exposure via protection from enzymatic degradation and efflux [11,12,13].

For oral administration, functionalizing a nanoparticle via mannosylation has the benefit of allowing for sustained input via intestinal lymphatic absorption, which may increase systemic exposure [14]. The intestinal lumen has microfold (M) cells in the follicular epithelium, covering immune response zones within the Peyer’s patch. These M cells express mannose receptors which facilitate endocytic trafficking of particles into the lymphatics [15,16,17]. Thus, the design of systems with a mannose presenting surface has become a potentially viable approach for enhancing the delivery of oral drug candidates [18,19,20]. However, engineering of a drug carrier for lymphatic trafficking must facilitate delayed intestinal release to promote lymphatic transit. Hydroxypropyl methylcellulose (HPMC), a semi-synthetic polymer variously used as a thickening, suspending, and emulsifying agent effectively stabilizes emulsions and facilitates controlled release of drugs [21,22,23]. Formulation of low bioavailability noscapine in a self-emulsifying drug delivery carrier stabilized by spray-drying with HPMC was therefore, predicted to delay systemic input and predispose to lymphatic transit and potential uptake.

In this study therefore, we hypothesized that noscapine, orally administered via a mannosylated HPMC-coated self-emulsifying carrier will be bioavailable and enhance tumor responsiveness to cisplatin. This we expected to result from a) slow noscapine release, b) increased lymphatic system transit, c) delayed/sustained systemic input, and d) increased plasma exposure, all potentially contributing to enhanced tumor sensitization. To test our hypothesis, we utilized a spray-dried mannosamine-HPMC matrix over a noscapine-loaded self-microemulsifying drug delivery system (SMEDD) to evaluate (a) the release rate, (b) transport across a colon cancer carcinoma cell (Caco-2) model, (c) pharmacokinetic plasma exposure in orally administered rats, and (d) tumor response to a combination regimen of noscapine and cisplatin in an orthotopic mouse model of chemorefractory H1650-induced non-small cell lung cancer (NSCLC).

## Materials and Methods

### Reagents

Noscapine hydrochloride, D-mannosamine hydrochloride, laminin, Accutase^®^, poly-D-lysine, epidermal growth factor, and fibroblast growth factor were from Sigma Aldrich (St. Louis, MO); Labrafil^®^ M1944 and Labrasol^®^ from Gattefosse (Paramus, NJ). Fetal bovine serum (FBS), RPMI-1640 media, DMEM:F12 base media and nitrogen supplement were from Life Technologies (Grand Island, NY). All other materials were of cell culture or analytical grade.

### Cell culture

H1650 cells, originally from the American Type Culture Collection (ATCC) were kindly donated by Dr. Srikumar Chellappan at the H. Lee Moffitt Cancer Center & Research Institute (Tampa, FL). Subcultures of H1650 cancer stem cells (CSCs)/side population (SP) cells were generated from H1650 parent/mixed population (MP) cells by fluorescence activated cell sorting with Hoechst 33342 staining as previously described [24]. Caco-2 and H460 cells were from the American Type Cell Culture (Manassas, VA). H1650 SP cells were maintained in DMEM:F12 supplemented with fibroblast growth factor (10 μg/mL), epidermal growth factor (10 μg/mL), and penicillin-streptomycin-neomycin (PSN, 2% v/v). H1650 MP cells were maintained in DMEM:F12 supplemented with 10% v/v FBS and 2% v/v PSN. Caco-2 cells and H460 cells were cultured in DMEM:F12 and RPMI-1640 respectively, each supplemented with FBS (10% v/v), PSN (2% v/v), non-essential amino acid solution (1.2% v/v) and HEPES (10 mM). All cell lines were maintained under carbon dioxide (5%) at 37°C.

### Preparation of solid microparticles

Optimized concentrations of oil (Labrafil^®^ M1944), surfactant (Tween^®^ 80) and co-surfactant (Labrasol^®^) were determined from tenary phase diagrams. The optimized composition consisted of an oil phase (Labrafil^®^ M1944; 63.5 mL, and Labrasol^®^; 9.39 mL) and an aqueous phase (Tween^®^ 80; 27.9 mL). Noscapine (5.0 g) was dissolved in chloroform (5.0 mL) and then dispersed in an oil phase comprising Labrafil M1944 and Labrasol (as the oil and co-surfactant respectively). The noscapine-oil dispersion was either emulsified in an aqueous phase (consisting of Tween^®^ 80and water) to form self-microemulsifying drug delivery systems (Nos-SMEDDs) or injected into a spray-dryer. Spray-drying was carried out by mixing the aqueous and oil phases within a heated (110°C) nitrogen atmosphere in the drying chamber of a Pulvis Mini-Spray GS-310 (Yamato Scientific America, USA). The aqueous phase (comprising Tween^®^ 80 and HPMC colloid (10% w/v), or Tween^®^ 80 and HPMC-Mannosamine HCl (1.0% w/v) colloid was inducted into a 1550 AutoJet Modular Spray System (Spraying Systems Co., Wheaton, IL) and the atomized mixture fed through channel 1 whereas the oil phase was simultaneously fed through channel 2 of the dual spray nozzle and atomized into fine oil droplets. Coating of oil droplets by colloidal aqueous phase occurs in the drying chamber under pulse flow with resultant noscapine self-emulsifying solid dispersible microparticles (Mann-Nos_SESDs or Nos_SESDs).

### Validation of nanoparticles and microparticles

Nos_SMEDDs and Mann-Nos_SMEDDs and Nos_SESDs and Mann-Nos_SESDs were characterized for size, polydispersity (PDI), zeta potential, and entrapment efficiency. Particle size and zeta potential of noscapine formulations were determined using a Nicomp380 ZLS (Particle Sizing Systems, Port Richey, FL). SMEDDs (10 μL), or SESDs (2.0 mg) were dispersed in double-distilled water to 4.0 mL in a cuvette and particle size, PDI, and zeta potential readings taken. Entrapment efficiency (EE) of SMEDDs and SESDs was determined by centrifugation of formulation using a vivaspin column with molecular weight cut-off (MWCO) of 10 kDa. The filtrate was collected and eluted over a C18 column by HPLC (see Bioanalysis Method). EE was estimated according to, EE (%) = [(W_n_—W_r_)/ W_n_] × 100, where, W_n_ = weight of Nos in SMEDDs or SESDs, and W_r_ = weight of Nos in filtrate. Determination of EE for SESDs was done by dissolving 10 mg of sample in 250 μL of acetic acid in a microcentrifuge tube. The sample was vortexed and centrifuged at 12,000 × *g* for 5 min. The aqueous phase was collected and 0.75 mL NaOH (4 M) added to precipitate Nos. The sample was centrifuged at 14,000 × *g* for 15 min and the supernatant discarded. The precipitate was dissolved in acetonitrile and eluted over a C18 column by HPLC. Determinations were done in triplicates for each formulation.

### Release of noscapine from microparticles

The dissolution of spray-dried noscapine microparticles (i.e. Nos_SESDs and Mann-Nos_ SESDs) was investigated under USP guidelines for delayed release dosage forms in a VanKel 7000 Dissolution System (Varian, Cary, NC) using the USP Dissolution Apparatus 1 basket method. The glass vessel was filled with 0.75 L of dissolution medium (i.e. 0.1 N HCl buffer, pH 2.0) and allowed to equilibrate at 37°C. Nos_ SESDs or Mann-Nos_ SESDs (0.5 g) was placed in the mesh basket and rotated at 50 rpm for 2 hr. Sampling (2.0 mL) was done from the dissolution medium at 5, 10, 15, 20, 30, 45, 60, 90 and 120 min. Volume correction was maintained by replacing the aliquot volume with an equal volume of fresh dissolution medium. A volume of 0.25 L of 0.2 M Na_3_PO4 was added to the dissolution medium and the pH adjusted to 6.8 at 120 min. Sampling and volume correction was continued at 4, 6, 20, and 24 hr. The samples were loaded onto a C18 column for HPLC analysis. Cumulative concentration was normalized against initial concentration and presented as Percent Drug Release vs Time. Determinations were done 2× in triplicates.

### Noscapine transport across Caco-2 monolayers

At 80% confluence, Caco-2 cells were detached with trypsin/EDTA and washed 2× with PBS. Cell suspensions (10^5^ cells per mL) were seeded in the apical compartment of a Corning^®^ Costar^®^ Transwell^®^ permeable support insert with polycarbonate membrane (0.4 μm pore size) in a 12-well plate format using 0.5 mL volume. Cells were maintained for 21 days with media changes on alternate days for 14 days and daily close to confluence. Formation and integrity of Caco-2 monolayer and tight junctions were monitored by transepithelial electrical resistance (TEER = 200–300 Ω∙cm^2^ using a Millicell^®^ ERS-2 Volt-Ohm Meter (Millipore, Billerica, MA). TEER below 190 Ω∙cm^2^ was discarded. Drug-free buffer (HBSS-HEPES) was added to the donor (pH 6.5) and acceptor (pH 7.4) compartments in a humidified (5% CO_2_), pre-warmed (37°C) incubator for 10 min for preconditioning. Sample solution was added to donor or acceptor compartment for flux:apical-basolateral or efflux:basolateral-apical estimates (i.e. A-B, B-A respectively), and drug-free buffer to counter compartment for sampling at 15, 30, 45, 60, 90, and 120 min. Transport was carried out on an orbital shaker (50 oscillations/min) to minimize the effect of an aqueous boundary layer. The directional transport of total noscapine (i.e. free Nos + SMEDD entrapped Nos) was estimated by liquid extraction with tert-methylbutyl ether and quantification by HPLC. The apparent permeability (P_app_) of Nos using this delivery system was computed at equilibrium (steady-state flux) according to: P_app_ (cm/s) = (ΔQ/Δt) × [(1/ Q_ss_) × (1/A)] × V_r_, where ΔQ/Δt = discrete difference in receiver mass per time interval (μg/s); Q_ss_ = mass of drug in donor chamber at steady state (μg); t = time (s); A = area of membrane insert (cm^2^); V_r_ = volume of solution receiver chamber (cm^3^). The net flux direction of total Nos across Caco-2 monolayers was estimated from an Efflux Ratio (ER) = (P_app_ B-A)/(P_app_ A-B). Experiments were repeated 2× and Papp estimates done in triplicates for each group.

### Bioanalysis method

Noscapine (Nos) stock solution (1.0 mg/mL) was prepared by dissolving Nos in acetonitrile. Working solutions of Nos were prepared by diluting the primary stock solution to obtain 2.5, 5, 10, 25.0, 50.0, and 100.0 μg/mL. Plasma calibration standards (0.4, 0.5, 1.0, 2.0, 10.0, and 20.0 μg/mL) were prepared in drug-naive rat plasma obtained from Sprague-Dawley (SD) rats. Rat plasma (160 μL) was spiked with Nos working solution (40 μL) and vortexed for 1 min. Nos was extracted by adding 100 μL tert-methylbutyl ether to the mixture and vortexing for 1 min. The organic phase was separated and the solvent evaporated. Quality control (QC) samples were prepared similarly to obtain plasma-drug concentration of 0.4, 1.0, and 10.0 μg/mL corresponding to low, intermediate, and high QC respectively. Samples were centrifuged at 12,000 × g for 15 min and the supernatant collected. Mobile phase consisting of 20 mM ammonium acetate (pH 4.5):water (65:35) was prepared and filtered using a 0.45 μm filter. Samples were injected (0.1 μL) into a Symmetry^®^ C18 column connected to a Waters^®^ HPLC under isocratic flow of 1.0 mL/min and detection carried out at λ_max_ of 232 nm. HPLC runs were done 3× for each sample and the same method was utilized for all analyses in this study.

### Ethics statement

The protocols for in vivo experiments were approved by the Institutional Animal Care and Use Committee (IACUC) of Florida A&M University.

### Pharmacokinetics in Sprague-Dawley rats

Sprague-Dawley rats, with mean weight of 250 g, were divided into 4 groups (n = 3) and given either a single dose of Noscapine (Nos) as an intravenous bolus at 2.0 mg/kg, or orally at 10 mg/kg, as either Nos in corn oil, Nos_SESDs or Mann-Nos_SESDs. The I.V. dose was prepared by dissolving Nos in a 0.1 mL volume of PBS (pH 4.5) and then administered by tail vein injection whereas oral doses were given by gavage. Blood samples were collected into heparinized tubes at 0.167, 0.5, 1, 4, 8, and 24 hr. The samples were centrifuged at 12,000 × *g* for 15 min, the plasma collected and analyzed as described (see Bioanalysis Method). A concentration-time plot of Nos was obtained and the data analyzed by simultaneous I.V. and Oral nonlinear regression of pooled data of all animals in each group to obtain pharmacokinetic parameters for a two compartment model with vascular (I.V.) extravascular (oral) input. The drug concentration was described by differential equations according to:

$$\begin{array}{l} \frac{dA_{\text{DOSE}}}{dt}=Ka•F•A_{\text{DOSE}}\;\\ \frac{dA_{\text{C}}}{dt}=Ka•F•A_{\text{DOSE}}\;+\;K_{21}•A_{\text{P}}\text{-}K_{\text{12}}•A_{\text{C}}\text{-}K\text{e}•A_{\text{C}}\\ \frac{dA_{\text{P}}}{dt}=K_{12}•A_{C}-\;K_{\text{21}}•A_{\text{P}}\\ A_{\text{C}}=C_{\text{P}}•V_{\text{C}}\\ K_{12}=Q\text{/}V_{\text{C}}\\ K_{21}=Q\text{/}V_{\text{P}}\\ Ke=CL\text{/}V_{\text{C}}\\ {\text{A}}_{\text{DOSE}}\text{(0) }=\text{ administered dose}\\ {\text{A}}_{\text{C}}\text{(0) }=\text{ 0} \end{array}$$

The Vss was the model estimated sum of volume of central (Vc) and tissue (Vp) compartments, whereas Cmax and Tmax were calculated with standard formulas using the pharmacokinetic parameters.

### H1650 SP and H1650 MP spheroid formation

H1650 SP or H1650 MP cell suspensions in serum-free media were seeded at 10^4^ cells per well in a 24-well Lipidure^®^-Coat Multi-Dish plate (NOF Corporation, Japan) up to 7 days for 3D culture, changing media on alternate days. Spheroid formation was observed on days 3, 5, and 7 in bright-field using an Olympus BX40 fluorescence microscope connected to a DP71 camera (Olympus, Japan) at 40 × magnification. The spheroid surface area was estimated using the ImageJ software (National Institutes of Health, Bethesda, MD). Results were expressed as mean surface area ± standard deviation of at least 3 replicates of two experiments each.

### Cell viability

The cell viability assay method was as previously described [25,26]. Briefly, H1650 MP, H1650 SP, or H460 cells were seeded in a 96-well format (1 × 10^4^ per well) and incubated for 16–18 hr. Treatment was carried out with different concentrations of cisplatin and/or noscapine and incubated for 72 hr. Cell viability studies for combinations of noscapine and cisplatin were done by varying the concentrations of cisplatin with fixed concentrations (5 and 20 μM) of noscapine and incubating for 72 hr post-treatment. The cells were washed with PBS 2× and fixed in 0.1 mL glutaraldehyde solution (0.025% w/v) and incubated at 37°C for 30 min. Glutaraldehyde was removed and 1 μL of (0.01% w/v) crystal violet solution was added and incubated for 15 min at room temperature. The plates were washed and air-dried followed by the addition of disodium hydrogen phosphate solution. The absorbance of the dissolved crystal violet was read at 540 nm and the cell viability calculated as a percentage of untreated controls. Determinations of cell viability were made at least 3× and the data presented as a plot of cell viability with log dose of treatment. The IC_50_ of each drug was computed where appropriate by regression and presented as mean IC_50_ ± SD. Similarly, the effect of 20 μM noscapine base in Nos_SESD, Mann-Nos_SESD, and their combination with different concentration of cisplatin on the cell 72-hr viability of H1650 SP cells was investigated. The 20 μM equivalent weight of Nos_SESD (26.70 μg) and Mann-Nos_SESD (25.80 μg) per ml of solvent (RPMI 1640) media were added to H1650 SP cells treated with and without different concentrations cisplatin to obtain a total noscapine base concentration of 20 μM in 200 μL final volume. The cells were incubated for 72 hr and analyzed for cell viability using the crystal violet assay. IC_50_ values representing mean ± SD of triplicate assays were estimated.

### Orthotopic H1650 NSCLC tumor model in Nu/Nu mice

Female Nu/Nu mice, six weeks old (Harlan, Indianapolis, IN) were placed under isoflurane-induced anesthesia under aseptic conditions. Left lateral chest was doused with iodine and cleaned with an alcohol swab. A small lateral incision (~5 mm) was made to the left chest in the plane of the left fore-limb just below the scapula. A cell suspension-filled B-D^®^ 1 mL latex free syringe connected to a 27-gauge Surflo^®^ winged infusion set was used to deliver an inoculum of 1 × 10^4^ H1650 SP (n = 29) or MP (n = 1) cells in 0.1 mL serum-free DMEM:F12, through the sixth intercostal space into the left lung. Incisions were closed with surgical skin clips and animals observed for full motor and cognitive recovery. Mice were maintained for 30 days for development of lung tumor verified by dissection of a mouse (bearing H6150 SP or MP tumors each) for anatomical observation and extraction of tumor lysate for western blot.

### Antitumor evaluation of noscapine in Nu/Nu mice

Animals were randomized into 7 groups (n = 4) of (i) untreated, (ii) Cisplatin, (Cis) (2 mg/kg/biweekly I.V.,), (iii) Nos_SESDs (300 mg/kg/day), (iv) Mann-Nos_SESDs (150 mg/kg/day), (v) Mann-Nos_SESDs (300 mg/kg/day), (vi) Mann-Nos_SESDs (150 mg/kg/day) + Cis (2 mg/kg/wk I.V.), and (vii) Mann-Nos_SESDs (300 mg/kg/day) + Cis (2 mg/kg/wk, I.V.,). Doses were selected based on previous studies in our laboratory [2,4,27,28]. Treatment was started on day 8 post-inoculation and continued for 14 days consisting of daily oral administration of Nos formulations and intravenous injections of Cis solution (biweekly as a single treatment or weekly in combination with Nos_SESDs). Cis solutions were made by dispersing Cis in normal saline under sonication and pH adjusted to 7.4 to obtain a 1.0 mg/mL suspension. To check for evidence of toxicity (weight loss >25%) or unreasonable tumor burden, the animals were weighed weekly. The mice were fed with food and water ad libitum. Mice were sacrificed under CO_2_-induced hypoxia and lung tumors resected. Tumor growth inhibition was estimated from lung weight and tumor weight.

### Statistical analysis

Analyses of nanoparticle characterization, spheroid formation, cell viability, western blot, lung and tumor weights were done using GraphPad Prism^®^ 5.0 (GraphPad Software, Inc.). Caco-2 permeation data analysis was done in Microsoft Excel. Pharmacokinetic data were analyzed using WinNonlin^®^ (Pharsight, Cary, NC). Differences between means were analyzed by unpaired t-test or One-way ANOVA, and considered significant at P < 0.05. Differences across group means were analyzed by One-way ANOVA. Comparisons between groups were analyzed by Tukey’s test. Data were presented as mean ± SD except otherwise stated.

## Results

### Spray-dried mannosamine-coated self-emulsifying carriers produce stable solid microparticles

Validity of in vivo data will be dependent on accurate characterization of stable particles. Optimization of the ternary system consisting of oil (Labrafil M1944), surfactant (Tween 80) and co-surfactant (Labrasol) resulted in a suitable composition by weight of 62.8% and 9.3% and 27.9% respectively. Stable SMEDDs resulted from mixing the components (1–3 parts) and dispersing in distilled water (7–9 parts). Results of SMEDD and SESDs characterization are presented in Table 1. Blank SMEDDS, Nos_SMEDDs, and Mann-Nos_SMEDDs were mondisperse (PDI of 0.260±0.002, 0.303±0.005, and 0.361±0.002 respectively) with mean hydrodynamic diameters of 230.91±6.33, 246.33±3.79, and 238.52±4.88 nm respectively. Negative surface charges of -16.50±0.82, -18.17±0.68, and -18.69±0.91 mV were recorded for Blank SMEDDs, Nos_ SMEDDs, and Mann-Nos_ SMEDDs respectively; all of which were within the range for nanoparticle emulsion stability. Entrapment efficiency of noscapine in Nos_ SMEDDs and Mann-Nos_ SMEDDs were 32.36±4.28% and 35.02±6.56% (n = 3) respectively, which enabled accurate calculation of doses. Particle size for Blank-SESDs, Nos_SESDs, and Mann-Nos_SESDs were 5.84±3.63, 6.42±5.03, and 6.60±3.16 μm respectively; the SESDs showed narrow distribution in particle size with polydispersity indices of 0.316±0.002, 0.293±0.003, and 0.266±0.003 respectively. Zeta potential was -12.18±1.32, -10.62 ± 0.83, and -12.40±1.61 mV for Blank SESDs, Nos_SESDs, and Mann-Nos_SESDs respectively. Entrapment efficiency of noscapine in Nos_SESDs and Mann-Nos_SESDs were 30.96±4.66 and 32.05±3.72% respectively.

**Table 1:** Characterization of Noscapine self-microemulsifying drug delivery (SMEDD) and self-emulsifying solid dispersible (SESDs) formulations for oral delivery.

**Sample**	**Particle Size(nm)**	**Polydispersity Index, PDI**	**Zeta-Potential, ζ (mV)**	**Entrapment Efficiency, EE (%)**
**Blank SMEDD**	230.91 ± 6.33	0.26 ± 0.002	-16.50 ± 0.82	-
**Nos-SMEDD**	246.33 ± 3.79	0.303 ± 0.005	-18.17 ± 0.68	32.36 ± 4.28
**Mann-Nos_SMEDD**	238.52 ± 4.88	0.361 ± 0.002	-18.69 ± 0.91	35.02 ± 6.56
**Sample**	**Particle Size(μm)**	**Polydispersity Index, PDI**	**Zeta-Potential, ζ (mV)**	**Entrapment Efficiency, EE (%)**
**Blank SESDs**	5.84 ± 3.63	0.316 ± 0.002	-12.18 ± 1.32	-
**Nos-SESDs**	6.42 ± 5.03	0.293 ± 0.003	-10.62 ± 0.83	30.96 ± 4.66
**Mann-Nos_SESDs**	6.60 ± 3.16	0.266 ± 0.003	-12.40 ± 1.61	32.05 ± 3.72

Noscapine was formulated in a self-microemulsifying drug delivery (SMEDD) system with and with mannosamine (Mann) and spray-dried to obtain self-emulsifying solid dispersible (SESD) particles. Particles were characterized for size, polydispersity, zeta potential, and entrapment efficiency as shown in Materials and Methods. Data are presented as mean ± SD of at least 3 analyses. Differences observed were not significant (one way ANOVA, P<0.05).

### Mannosylated microparticle retains 50% of oral noscapine through 6 hours

**Fig 1:**
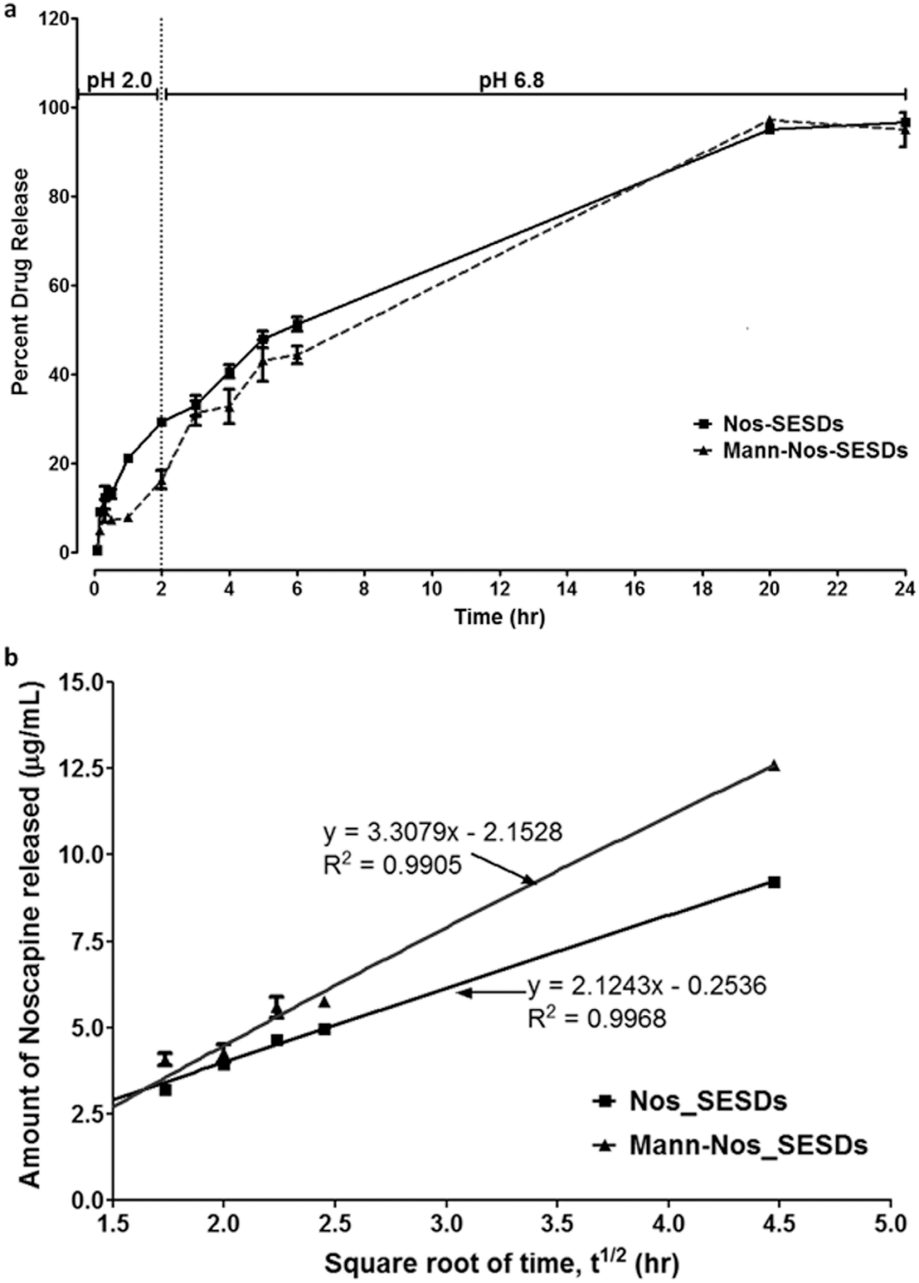
Mannosylated microparticle retaines 50% of oral noscapine in 6 hours. (a) The dissolution rate and release of total noscapine from HPMC-coated spray-dried Nos_SMEDD and Mann-Nos_SMEDD are shown in simulated gastric (pH 2.0) and intestinal (pH 6.8) buffers from 5 min to 24 hr. Results were presented as mean percent of Nos release vs time. (b) Kinetic release of Nos_SMEDD and Mann-Nos_SMEDD showing Higuchi relationship of drug amount released from solid microparticle into dissolution buffer against square root of time. Straight line equation obtained by linear regression. Both formulations were linear with R^2^ ranging between 0.9905 and 0.9968. Data represents 6 replicates of experiments repeated 2× and differences were not considered significant.

The aqueous release characteristics of spray-dried Nos_SESDs and Mann-Nos_SESDs were investigated using the USP Apparatus 1 method for delayed-release dosage forms under gastric and intestinal conditions (i.e. p*H* 2.0 and 6.8 respectively) (Fig 1). At gastric pH, a maximum of 29.4% of Nos_SESDs versus 16.4% of Mann-Nos_SESDs was observed at the end of 2 hr (Fig 1a). At intestinal pH, 51.3% and 44.4% of noscapine was released from Nos_SESDs and Mann-Nos_SESDs respectively within the next 4 hr (Fig 1a). Noscapine release from both formulations was almost 100% at 24 hours. The data were fitted into zero-order, first-order, and Higuchi kinetic models for time predictability, and showed the highest fidelity to the Higuchi relationship where Q(Mass) = K_H_ × t^1/2^. A plot of concentration of drug released against square root of time produced linear diffusion profiles for Nos_SESDs and Mann-Nos_SESDs described by the equations Q = 2.124t^1/2^–0.254 (R^2^ = 0.997) and Q = 3.308 t^1/2^–2.153 (R^2^ = 0.991) respectively (Fig 1b).

### Mannosylated microparticle saturates noscapine transcytosis

**Fig 2:**
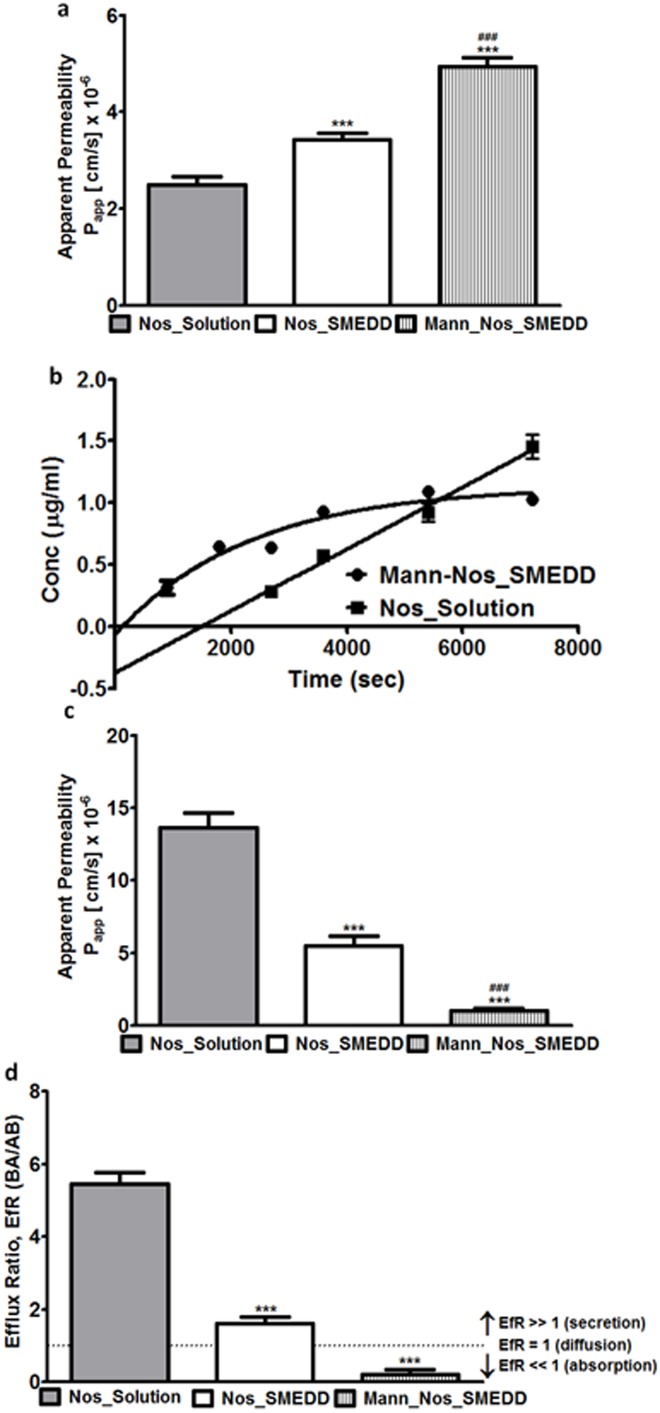
Mannosylated microparticle saturates noscapine transcytosis. Transport of Nos formulations across Caco-2 monolayers. (a) Absorptive apparent permeability (Papp) of Nos solution (2.50±0.17 × 10^−6^ cm/s), Nos_SMEDD (3.43±0.13 × 10^−6^ cm/s), and Mann-Nos_SMEDD (4.94±0.18 × 10^−6^ cm/s). (b) Concentration vs time plot of Nos solution and Mann-Nos_SMEDD across Caco-2 monolayers showing linear pseudo zero order transport and apparent first order transport, respectively. (c) Secretory apparent permeability (Papp) of Nos solution (13.66±1.01 × 10–6 cm/s), Nos_SMEDD (5.50±0.65 × 10–6 cm/s), and Mann-Nos_SMEDD (1.03±0.15 × 10–6 cm/s). (d) Efflux ratios of total Nos from Nos solution, Nos_SMEDD, and Mann-Nos_SMEDD. Efflux ratios were estimated by dividing the secretory apparent permeabilities by the corresponding absorptive apparent permeability for each formulation. Results are presented as means ± SD. Results are from triplicates of at least two experiments per group. Differences in means were analyzed by unpaired t-test; P<0.05 were considered significant (***P < 0.0001, vs Nos solution; ^###^P < 0.0001, vs Nos_SMEDD).

The permeation results across caco-2 monolayers are shown in Fig 2. The estimated apparent permeability (Papp) for total noscapine from Nos_Solution, Nos_SMEDD, and Mann-Nos_SMEDD, revealed that the constant for transcytosis was highest using the mannosylated formulation. The apical/donor (pH 6.5) to basolateral/acceptor (pH 7.4) permeability of total noscapine was low in Nos_Solution (2.50±0.17 × 10^−6^ cm/s) compared to Nos_SMEDD (3.43±0.13 × 10^−6^ cm/s), and Mann-Nos_SMEDD (4.94±0.18 × 10^−6^ cm/s). A plot of concentration vs time for total noscapine in Nos solution and Nos_SMEDD were approximately linear therefore, estimation of their Papp was based on the assumption of rate saturation and zero-order kinetics. The time course of total noscapine transport from Mann-Nos_SMEDDS followed first-order kinetics with apparent receptor saturation (Fig 2b). The differences in the secretory transport of total Nos was in the order of Nos solution (13.66±1.01 × 10^−6^ cm/s) >>> Nos_SMEDDS (5.50±0.65 × 10^−6^ cm/s) >> Mann-Nos_SMEDDS (1.03±0.15 × 10^−6^ cm/s) (Fig 2c). In parallel order, efflux ratios (ER) followed the hierarchy of Nos solution (5.46) >>> Nos_SMEDD (1.61) >> Mann-Nos_SMEDD (0.21±0.13), indicating the mannosylated microparticle was least subject to efflux (Fig 2d).

### Mannosylated microparticles have delayed absorption and higher bioavailability

**Fig 3:**
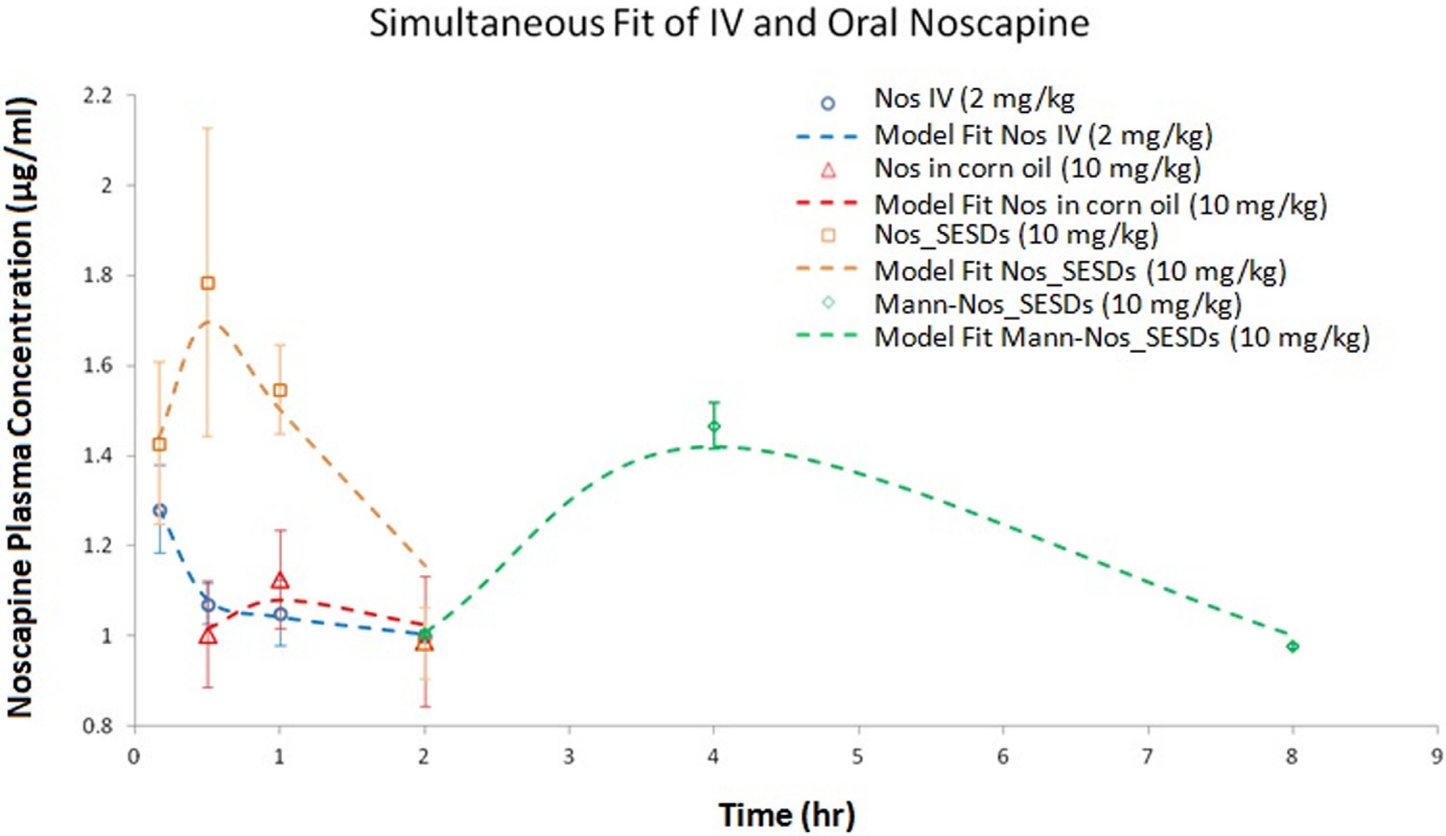
Mannosylated microparticles have delayed absorption and higher bioavailability. Fig shows pharmacokinetic profiles of intravenous Nos solution and oral formulations in Sprague-Dawley rats. Rats were fasted overnight and given a single intravenous bolus Nos (2 mg/kg), or 10mg/kg orally as Nos in corn oil, Nos_SMEDD, and Mann-Nos_SMEDD. Plasma samples were obtained and analyzed for the concentration of Nos as described in Materials and Methods. Data are presented as means ± SD of plasma concentration of Nos (μg/mL) with time (n = 3 per group).

Plasma drug concentration-time plot for each formulation is shown in Fig 3. Pharmacokinetic parameters were obtained by nonlinear regression of plasma concentration vs time data points using two-compartmental analyses of intravenous and oral input of noscapine are shown in Table 2. Intravenous bolus injection of Noscapine solution at 2 mg/kg was distributed into the peripheral/tissue compartment at a rate of 3.867 h^-1^ (k12); the rate of drug transport from the peripheral compartment into the central compartment (k21) and rate of drug elimination were 4.677 h^-1^ each. Total volume of drug distribution at steady state (Vss) was 1856.442 mL, and the volume of distribution in the central (Vc) was 1016.209 mL. The rate of systemic clearance (Cl) was 46.524 mL/h with an elimination half-life (T1/2) of 15.137 h and area under the concentration-time curve (AUC_0-4_) of 3.925 μg·h/mL. Oral formulation of noscapine in corn oil at a dose of 10 mg/kg was characterized by an absorption rate (ka) of 5.161 h^-1^ with a half-life of drug absorption, T1/2, abs of 0.134 h. The rate of drug elimination, kel was 0.301 h^-1^ with a terminal half-life, T1/2 elimination of 2.302 h. The maximum plasma drug concentration, Cmax was 0.945 μg/mL and the time to reach maximum concentration, Tmax was 0.585 h. The area under the curve was 3.812 μg·h/mL and the absolute bioavailability was 19.424%. At 10 mg/kg oral dose, Nos_SESD the ka, T1/2 abs, kel, and T1/2 elimination were 4.729 h^-1^, 0.147 h, 0.160 h^-1^, and 4.331 h respectively; the Cmax, Tmax, AUC, and absolute bioavailability were 1.627 μg/mL, 0.742 h, 5.191 μg·h/mL, and 26.451% respectively. The ka, T1/2 abs, kel, and T1/2 elimination of oral Mann_Nos_SESD were 0.636 h^-1^, 1.090 h, 0.101 h^-1^, and 6.861 h respectively; the Cmax, Tmax, AUC, and absolute bioavailability were 1.308 μg/mL, 3.439 h, 7.274 μg·h/mL, and 36.927% respectively.

**Table 2:** Pharmacokinetic profiles of noscapine formulations in rats.

PK Parameter	Nos IV	Nos_corn oil	Nos_SESDs	Mann_Nos_SESDs
**Dose (μg/kg)**	2,000	10,000	10,000	10000.000
**Vc (mL)**	1016.209	-	-	-
**Cl (mL/h)**	46.524	-	-	-
**ka (h**^**-1**^**)**	-	5.161	4.729	0.636
**T1/2, abs (h)**	-	0.134	0.147	1.090
**kel (h**^**-1**^**)**	0.046	0.301	0.160	0.101
**k12 (h**^**-1**^**)**	3.867	-	-	-
**k21 (h**^**-1**^**)**	4.677	-	-	-
**T**_**1/2**_**, elimination (h)**	15.137	2.302	4.331	6.861
**Tlag (h)**	-	-	-	1.760
**Tmax (h)**	-	0.585	0.742	3.439
**Cmax (μg/mL)**	-	0.945	1.627	1.308
**AUC (μg·h/mL)**	3.925	3.812	5.191	7.247
**Bioavailability (%)**	-	19.424	26.451	36.927

Sprague-Dawley rats (n = 3, average weight of 0.25 kg) were administered noscapine by intravenous injection (Nos_IV, 2 mg/kg); and noscapine dispersion in corn oil (Nos_Corn oil, 10 mg/kg), noscapine self-emulsifying solid dispersions (Nos_SESDs, 10 mg/kg) and mannosamine-coated noscapine self-emulsifying solid dispersions (Mann-Nos_SESDs, 10 mg/kg) by oral gavage. Plasma samples were taken at different time points and the concentration of noscapine was determined using by HPLC. Plasma concentration-time course data were analyzed using a two-compartment model to obtain pharmacokinetic parameters. Data is presented as mean ± SD (n = 3). Vc: Volume of distribution in central compartment; Cl: Clearance; ka: rate constant of drug absorption; k12: rate constant of drug distribution; k21: rate constant of drug transport from tissue/peripheral compartment to central compartment; kel: rate constant of drug elimination; T1/2: elimination half-life; Tlag: lag time of drug absorption; Tmax: time to reach maximum plasma drug concentration; Cmax: maximum plasma drug concentration; AUC: area under the plasma concentration-time curve.

### H1650 SP cells are chemoresistant compared to H1650 MP and H460 cells

**Fig 4:**
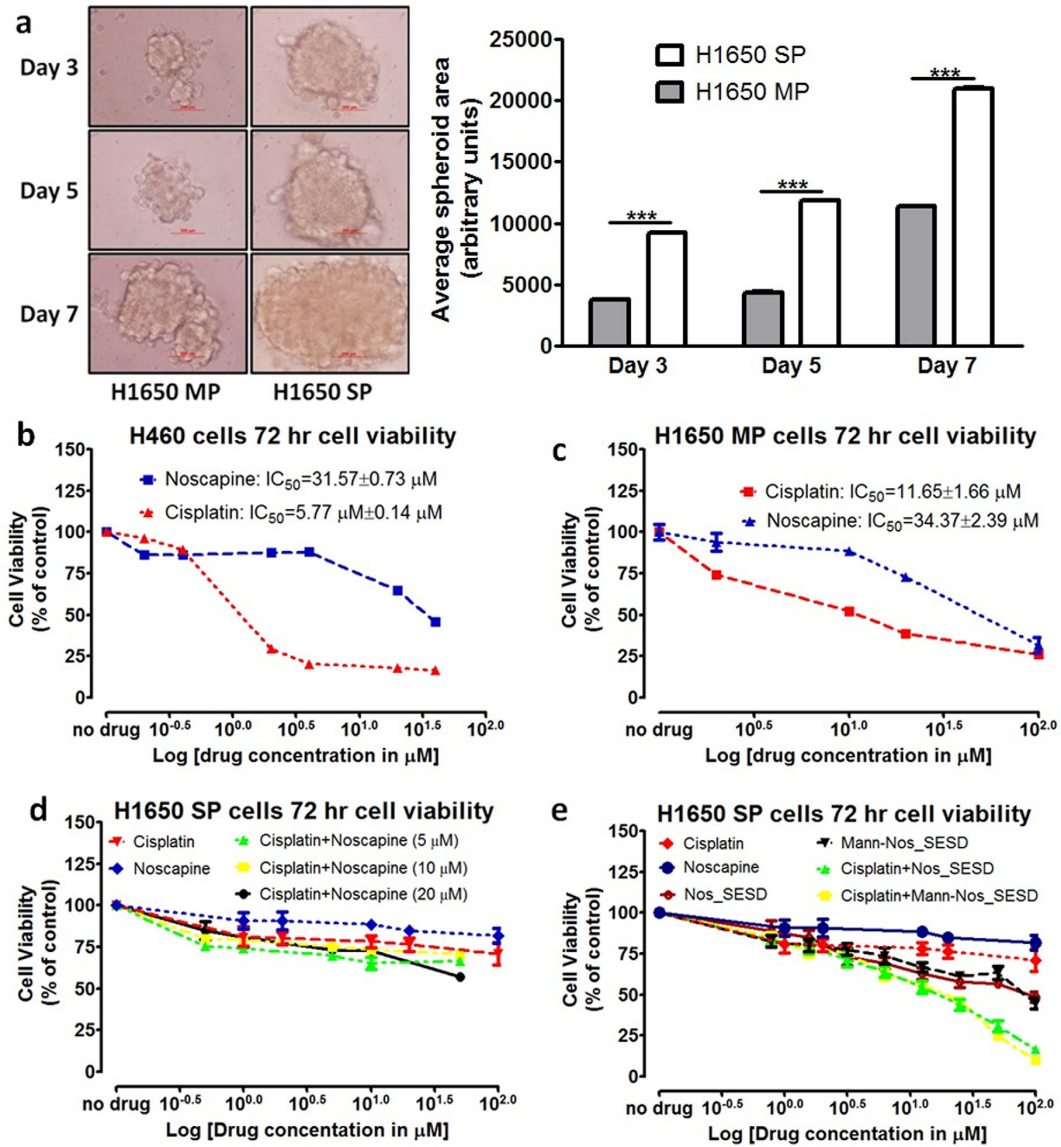
Chemoresistant H1650 SP cells are sensitized to Cisplatin by Nos_SESDs and Mann-Nos_SESDs. (a) H1650 SP cells showed higher spheroid formation potential compared to H1650 MP cells (***P<0.0001). (b-d) Anticancer effects of Noscapine (Nos) and Cisplatin (Cis) determined by cell viability studies in (b) H460, (c) H1650 MP, and (d) H1650 SP cells after 72 hr incubation; single and combination treatments of Cis, Nos, and Cis with fixed concentrations (5, 10, and 20 μM) of Nos after 72 hr incubation demonstrated no significant anticancer effects. (e) The marginal decreases in H1650 SP cell viability following treatment with Nos_SESDs and Mann-Nos_SESDs are significantly enhanced when giving in combination with Cis. Results were presented as cell viability (percent of control) against log concentration as triplicates of at least two different experiments.

To establish the resistance potential of H1650 SP cells used in our model, we compared the abilities of untreated H1650 SP and H1650 MP cells to form spheroids in matrix-coated Lipidure^®^ plates as well as its comparative response to Cisplatin (Cis) and Noscapine (Nos) (Fig 4). Fig 4a presents average spheroid surface area (in arbitrary units) for H1650 SP and H1650 MP at Day 3 (373.57±4.71 and 923.40±1.54 respectively), Day 5 (436.7±3.68 and 1183.80±4.19 respectively), and Day 7 (1132.93±1.12 and 2096.40±9.94 respectively). These results represented percentage fold differences in spheroid growth between H1650 SP and H1650 MP cells of 247.18%, 271.04%, and 185.04% at Days 3, 5, and 7 post-seeding, respectively.

### Noscapine increases H1650 SP sensitivity to cisplatin by up to 43% (P<0.05)

Noscapine (Nos) and cisplatin (Cis) each exhibited dose-dependent inhibition of H460 and H1650 MP 72 hr cell viabilities (Fig 4b and 4c). Nos and Cis displayed reciprocal potency against chemosensitive H460 cells (IC_50_ 31.57±0.73 μM and 5.77±0.14 μM, respectively) and the H1650 MP cells (IC_50_ 11.65±1.66 μM and 34.37±2.39 μM, respectively), as expected. However, chemoresistant H1650 SP cells were unresponsive to either Nos or Cis through 20 μM (Fig 4d); however, Cis efficacy against H1650 SP increased marginally following combination treatment with Nos solution at 5, 10, and 20 μM (Fig 4d).

### H1650 SP cells show sensitivity to Nos_SESDs and Mann-Nos_SESDs

As shown in Fig 4e, the anticancer efficacy of single treatments with Nos_SESDs and Mann-Nos_SESDs and their combinations with different concentrations of Cis were investigated in H1650 SP cells using 20 μM concentration of Nos base for each formulation. The IC_50_ of Nos base in Nos_SESDs and Mann-Nos_SESDs were down to 90.43±10.85 μM and 85.52±6.48 μM compared to Nos solution (IC_50_ > 100 μM). In combination with Cis solution, 20 μM Nos base in Nos_SESDs and Mann-Nos_SESDs decreased the IC_50_ of Cis to 17.72±3.05 μM and 20.08±4.76 μM respectively.

### Mannosylated noscapine microparticles enhance cisplatin activity in NSCLC

**Fig 5:**
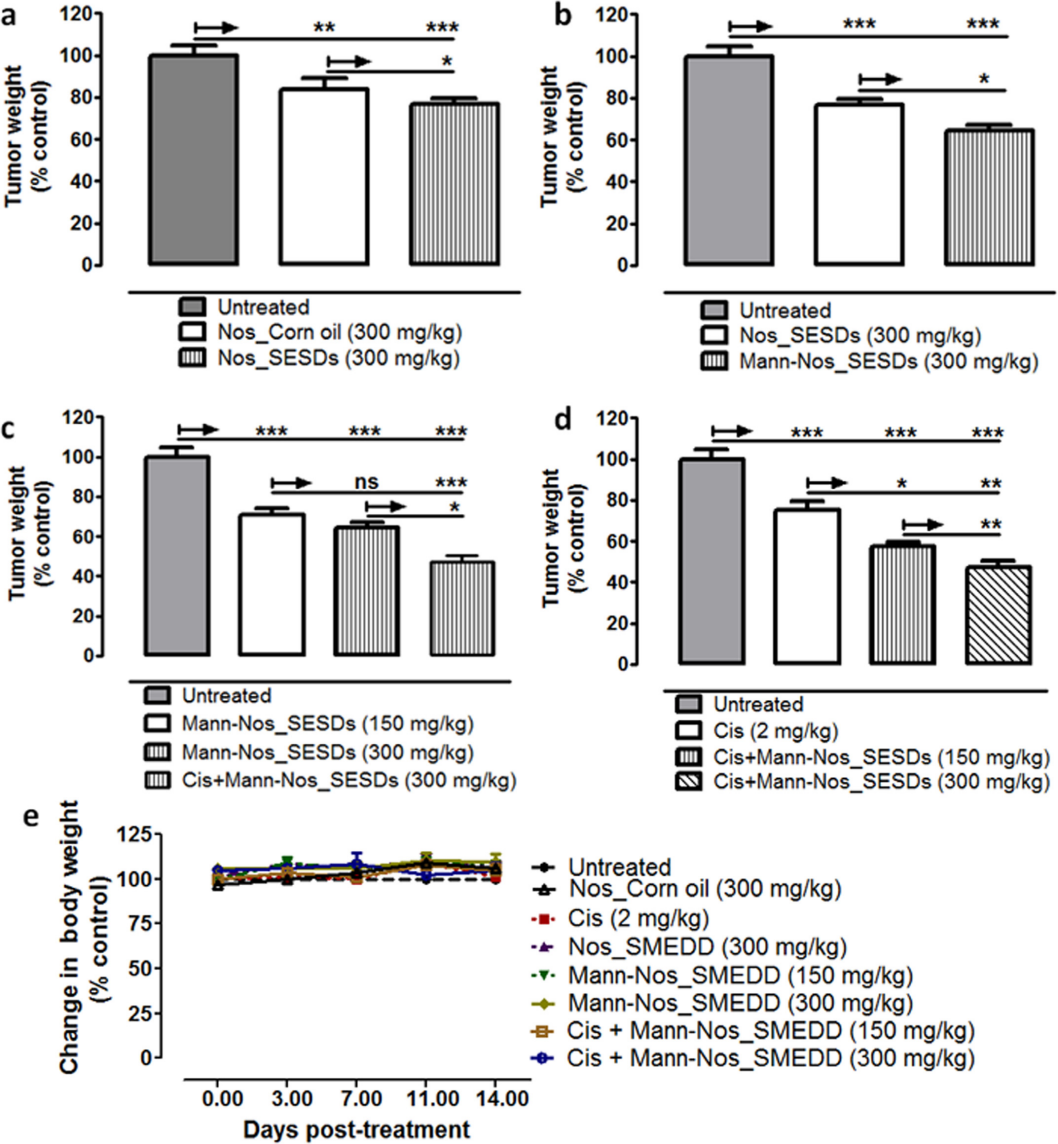
Mannosylated noscapine microparticles enhance cisplatin sensitivity in NSCLC. Panel displays anti-tumor effects of Nos-SMEDD and Mann-Nos_SMEDD in nu/nu mice bearing orthotopic lung tumors, alone and in combination with Cisplatin. Fig 5a shows the effect of oral noscapine and the solid microparticle. Fig 5b shows the effect of mannosylation over the solid microparticle. Fig 5c shows dose sensitivity of noscapine. Fig 5d shows the effect of the mannosylated microparticle on cisplatin response,and Fig 5e shows potential toxicity of treatment with respect to mouse body weight. Results are presented at percent means ± SD normalized against untreated control group (n = 4 per group).Differences in means were analyzed by One-way analysis of variance (P<0.05), followed by Bonferroni post-test with 90% confidence intervals (P<0.1). Significance was reached at *P<0.1, **P<0.01, and ***P<0.001.

The data for tumor inhibition were analyzed on the basis of effects of (a) formulation, (b) mannosylation, (c) dose sensitivity, and (d) combination of Cis with Nos. Compared to the untreated control group, Nos_Corn oil (300 mg/kg) and Nos_SESDs (300 mg/kg) inhibited lung tumor growth by 16% (P<0.001) and 23% (P<0.001) revealing that the particulate formulation was more effective than solution (Fig 5a). The mannosylated formulation however, at 300 mg/kg, inhibited tumor growth by 36% compared to the untreated control (P<0.001); a 1.6 fold increase compared to Nos_SESDs (P<0.05) (Fig 5b). With dose sensitivity comparison between 150 mg/kg and 300 mg/kg of Mann-Nos_SESDs, and the latter was about 24% more effective in inhibiting lung tumor growth (P>0.05) (Fig 5c). Lastly, the combination of intravenous Cis (2 mg/kg, per week) and Mann-Nos_SESDs at 150 mg/kg and 300 mg/kg was more effective than Cis alone (2 mg/kg, 2× per week), resulting in 18% (P = 0.029) and 28% (P = 0.004) more tumor inhibition, respectively (Fig 5d). Mice body weights were taken 2× per week to monitor toxicity of treatments. Changes in body weight were in the range of 3.65 to 10.12% compared to untreated controls, and were not considered significant indicators of toxicity, or animal stress due to tumor burden (P>0.05) (Fig 5e).

## Discussion

This study supports a proposed paradigm where tumor sensitivity of chemoresistant cells may be effectuated by sufficient tumor exposure. The so-called cancer stem cells or side populations represent a niche of intractable tumors due to their self-renewability coupled with potential for drug efflux, drug metabolism, and enhanced metastasis all culminating in increased chemo-refractoriness [25]. In order to inhibit tumor progression and further metastases, drugs have to be able to kill or diminish these niches. Here we demonstrate a formulation approach for enhancing systemic uptake of noscapine for sensitization of CSC-derived chemo-refractory lung tumor.

Noscapine suffers from the fate of liver and extra-hepatic metabolizing enzymes which diminish its guarantee of enhanced systemic levels needed to elicit antitumor activities [9]. In particular, for drugs with short half lives which may be cell-cycle dependent, the translational success of such orally administered compounds without resorting to continuous IV infusions, is known to be easily compromised [29]. We fabricated noscapine in a stable microparticle to delay intestinal release and promote lymphatic transit and residence. The increased lymphatic residence was anticipated to promote lymphatic uptake and limit hepatic input and first-pass metabolism; resulting in an increase in absolute bioavailability. This also has potential for delaying systemic input due to the slow rate of lymphatic transport, which increases plasma exposure and bioavailability. Enhanced absorption following oral administration was achieved by engineering a spray-dried noscapine formulation of a HPMC-coated lipid-based self-emulsifying solid dispersed system (SESDs), with mannosamine [30]. The mechanism of drug release from the HPMC matrix core may be summed up as pure diffusion without erosion of the matrix in this study, and mannosylation was observed to cause an increase in the Higuchi rate constant, when compared to the non-mannosylated microparticle (Fig 1b) [31].

In addition to a functional release rate, it was promising to observe a very low efflux ratio (Fig 2) for mannosylated microparticles compared to the non-mannosylated system, as efflux is one mechanism of drug resistance in lung cancer. Intriguing however, was the significant increase in apparent permeability (Papp) of total Nos from Mann-Nos_SESDs compared to Nos_SESDs; which we attributed to differences in endocytic vs transcytotic uptake [30]. We did not assume mannosylation prevents the transcytotic pathway afforded to microparticles, however the likely contribution of receptor-mediated endocytosis was supported by a first-order concentration-time curve for Mann-Nos_SESDs presumably due to increased receptor saturation with time (Fig 2b). As such, the permeability for the mannosylated formulation was estimated at the initial donor concentration whereas for the other formulations, it was derived using the average rate of transport at presumed steady state (i.e. linear portion of the concentration vs. time relationship). Although receptor-mediated uptake can display faster initial transport rates, there may be reduced total absorption in fasted models (as used in this study) affecting the bioavailability [32]. The analysis of total Nos from Mann-Nos_SESDs preparations in vitro, nonetheless allowed us to conclude enhanced transport rates across Caco-2 cells (Fig 2a) compared to noscapine in solution which we attributed to increased surface recognition of mannosamine.

The in vitro release of noscapine from microparticles could not be correlated with the rat pharmacokinetic profile because the bioanalysis method quantified total noscapine available in plasma. The pharmacokinetic study showed 40–90% increases in AUC for particulate formulations compared to corn oil, and a 40% increase in Mann-Nos_SESDs absolute bioavailability compared to Nos_SESDs. It is known that oral noscapine bioavailability behaves nonlinearly (i.e. increases) at higher doses [8], consistent with saturating first pass. We utilized a low dose, 10mg/kg in lieu of 300mg/kg, in this study to ensure the PK response observed is in the pharmacokinetic linear range. Hence, increases in bioavailability observed in this study may be attributed to the formulation without contributions from molar-dependent dynamics in absorption.

The plasma concentration vs time curve of Nos following intravenous bolus administration exhibited a biexponential decline (Fig 3) and was fitted into a two-compartmental model to generate the pharmacokinetic parameters (Table 2). Notably, Mann-Nos_SESDs exhibited delayed systemic appearance (lag time of 1.76) and slower absorption (7- and 8-fold decrease in rate of absorption respectively) compared to Nos in corn oil or Nos_SESDs. The delayed rate of drug elimination resulting in a higher elimination half-life of Mann-Nos_SESDs is indicative of increased systemic exposure, thereby, supporting our hypothesis of particulate protection from hepatic degradation as observed with a 40% higher area under the plasma concentration vs time curve and absolute bioavailability of Mann-Nos_SESDs compared to Nos_SESDs. This, we believe was afforded by lymphatic exposure considering the faster permeation observed in Caco-2 cells (Fig 2). The slow input (longer T1/2abs), slow elimination, and high systemic bioavailbility of Mann-Nos_SESDs was hypothesized to translate into better anticancer activities attributed to extended plasma exposure, and consequently greater coverage of the cell-cycle distribution [33].

Sphere or colony formation is a hallmark of tumorigenic potential; and this potential is increased in chemoresistant and highly metastatic cancer cells [34]. Our data supported a highly tumorigenic H6150 SP/CSC niche compared to their mixed lineage H1650 MP counterparts (Fig 4a). This was consistent with the decreased susceptibility of H1650 SP cells (Fig 4d) to Cisplatin (Cis) or Noscapine (Nos) compared to H1650 MP and the positive control H460 cells (Fig 4b and 4c). Due to their very high resistant nature, the resistance of H1650 SP cells to the antimitotic noscapine in solution was expected. The marginal increase in anticancer efficacy of solutions of Cis and Nos combination treatment was an encouraging prospect for a Nos formulation approach to sensitizing H1650 SP cells to chemotherapy. Significantly, in vitro combination treatment with either Nos_SESDs and Mann-Nos_SESDs and Cis demonstrated synergism in inhibiting H1650 SP cell proliferation (Fig 4e). The lack of significant differences in the in vitro anticancer effects of single treatments with Nos_SESDs and Mann-Nos_SESDs or with their combinations with Cis can be reasonably justified by the presumably equivalent drug exposures of Nos from both formulations.

The noscapine tumor response was likely dependent on enhanced plasma exposure as shown by tumor regression comparisons in Fig 5b and 5c, respectively. While our search of literature did not reveal any comparative studies of noscapine in an orthotopic mouse model of highly chemo-refractory lung cancer stem cell tumors, our response data for the 2-week Mann-Nos_SESDs regimen compared well with our previous reports of orally administered Nos in mice bearing H460 cell xenografts over a 4-week period [2]. We anticipate that an extended treatment period would afford a much higher antitumor efficacy over what is observed in the present study. However, the significant reduction in cancer stem cell lung tumors in the Cisplatin with Mann-Nos_SESDs group underscores the rationale for a combination therapy approach in treatment chemo-refractory tumors [27,35].

In this study, and in keeping a previous study [36], no significant advantage in terms of tumor regression was afforded with a high dose (300mg/kg) of noscapine formulated as SESDs. This was possibly due to the highly resistant nature and low sensitivity of this chemoresistant model, the short duration of treatment, or a combination thereof. In support of our conclusion of tumor responsiveness, we rely on the finding of increased in vivo tumor regression in H1650 SP cells and lung tumors.

## Conclusion

The limited toxicity of noscapine is an ideal characteristic for exploiting its cytotoxic effect. Barring extensive metabolism of noscapine by hepatic enzyme activities, high therapeutic outcomes could be realized with low oral doses and prolonged exposure. The potential for lymphatic uptake via mannosylation is applicable for compounds which present with systemic exposure challenges, such as poor solubility, low bioavailability and short half lives. By engineering a mannosylated spray-dried self-emulsifying solid microparticle formulation of noscapine, we facilitated delayed systemic input, increased bioavailability, and enhanced therapeutic. Moreover, the approach utilized herein resulted in pharmacokinetic and antitumor responses suitable for inhibiting chemo-refractory lung tumors with reduced toxicity.
